# Mobility, ecotoxicity, bioaccumulation and sources of trace elements in the bottom sediments of the Rożnów reservoir

**DOI:** 10.1007/s10653-021-00957-4

**Published:** 2021-05-10

**Authors:** Magdalena Szara-Bąk, Agnieszka Baran, Agnieszka Klimkowicz-Pawlas, Joanna Tkaczewska, Barbara Wojtasik

**Affiliations:** 1 Department of Agricultural and Environmental Chemistry, University of Agriculture in Krakow, al. Mickiewicza 21, Krakow, Poland; 2 Department of Soil Science Erosion and Land Protection, Institute of Soil Science and Plant Cultivation – State Research Institute, Czartoryskich 8, 24-100, Puławy, Poland; 3 Department of Animal Product Processing, University of Agriculture in Krakow, Krakow, Poland; 4 Department of Genetics and Biosystematics, University of Gdańsk, Gdańsk, Poland

**Keywords:** Trace elements, Fractions, Risk assessment, Mussels, Biotests, Bottom sediments

## Abstract

The aim of the study was to use of geochemical, chemical, ecotoxicological and biological indicators for a comprehensive assessment of ecological risks related to the mobility, ecotoxicity and bioavailability of trace elements in the bottom sediment of the Rożnów reservoir. The study found three elements deserving attention in the sediments: cadmium, nickel and chromium. Cadmium proved to be the most mobile and bioavailable, although the total cadmium content and geochemical indicators did not reveal any risk to organisms. Geochemical indicators showed that the sediments are contaminated with nickel and chromium, but both elements had a low bioaccumulation factor. Fractional analysis also revealed relatively low mobility of Cr and Ni and a higher potential risk of bioavailability for nickel. Most of the tested sediment samples had low toxicity in relation to the tested organisms. For H. incongruens, 11% of the samples were non-toxic, 50% of the samples had low toxicity, and 39% of the samples were toxic. For A. fischeri, no toxicity was found in 7% of the samples, low toxicity in 76% of the samples and toxicity in 17% of the sediment samples. The As, Cd, Cu content in the F1 fraction correlated significantly positively with the content of these metals in mussel tissues. Both biotesting and chemical analysis can reveal a potential risk to aquatic organisms. For a real assessment of the ecological risks associated with trace elements, it is necessary to use bioindicators taken from the environment and exposed to trace elements in situ.

## Introduction

Contamination with trace elements is the result of natural processes in the environment (e.g., weathering of rocks) as well as anthropogenic activity (Sarkar et al., 2014). The mobility and bioavailability of trace elements depend on the chemical form in which they occur in the environment. The most mobile is elements of anthropogenic origin in ionic and carbonate form. In turn, elements bound to silicates and primary minerals are characterized by limited mobility (Klink et al., 2019; Morillo, 2008). The analysis of total trace elements does not provide sufficient information to determine the risks associated with mobility and potential bioavailability, and therefore, methods allowing to determine individual metal fractions are used (McCready et al., 2010). The BCR sequential extraction procedure is the most common method here (Gleyzes et al., 2002; Nieto et al., 2007; Rosado et al., 2016) and involves increasing the extraction power from weak acids through reducers and oxidizers to strong mineral acids in order to dissolve successive trace element fractions (Bacon & Davidsion 2008; Sarkar et al., 2014). Sequential chemical extraction by the modified BCR method allows to extract four fractions of trace elements: F1–exchangeable, easily soluble in acidic environment, characterized by the most unstable binding with sediments and therefore the most potentially available; F2–reducible, bound to Fe and Mn oxides, available when the oxygen conditions change; F3–oxidizable, bound to organic matter and sulfides, released under oxidative conditions; F4–residual, elements permanently bound to minerals, unavailable fraction (BCR, 2001; Sarkar et al., 2014; Baran et al., 2019a). However, it should be emphasized that the knowledge of the total metal content and fractions obtained through chemical analyses only allows for the assessment of potential hazards associated with trace elements in bottom sediments. A useful tool for the actual assessment of the risk related to the presence of trace elements in the bottom sediment, i.e., their bioavailability, ecotoxicity, is biological methods including biotests and bioindicators (Apitz, 2011; Hise et al., 2020). Biotests allow for the assessment of toxicity associated with the presence of contaminants in bottom sediments, while bioindicators enable the assessment of the level of bioavailability and bioaccumulation of a given compound (Apitz, 2011; Shirneshan et al., 2013; Vignati et al., 2019; Hise et al., 2020). In the case of aquatic ecosystems, bottom sediments are the main sorbents of contaminants whose concentrations in them reach much higher values than in water (Ashokkumar et al., 2009; Rosado et al., 2016). A change in environmental conditions, such as pH or redox potential, can lead to the release of elemental contaminants from sediments into the water (Horsfall & Spiff, 2002; Morillo et al., 2008; Zoumis et al., 2001). This may have an adverse effect on benthic organisms due to their exposure to contaminants and ability to accumulate the bioavailable fraction of trace elements (Apitz, 2011). Therefore, benthic invertebrates are an important factor in the assessment of bioaccumulation and a link in the transport of contaminants up the trophic chain (Birch & Apostolatos, 2013; Burgos & Rainbow, 2001; Morillo et al., 2008). From all benthic organisms, mussels seem to be a good biological indicator of the mobility and bioavailability of trace elements in aquatic ecosystems, as they are filter feeders and live a sedentary lifestyle (Heroika et al., 2018; Klink et al., 2019; Honda & Suzuki, 2020). Biotests are ecotoxicological methods which can be used to assess the potential biological response in environmental samples (Kuczyńska et al., 2005). An important factor in the use of biotests is the selection of an appropriate battery of test organisms. It is important that organisms belong to different taxonomic groups and represent different links in the trophic chain (Szara et al., 2020a, 2020b). According to current data, sediment chemical analyses should be complemented by ecotoxicological studies, as the large number of potentially toxic compounds in sediments makes chemical assessment time-consuming, costly and often impossible. In addition, neither bioavailability nor interaction between substances can be investigated by chemical methods (Wadhia & Thompson, 2007; Baran & Tarnawski, 2015; Cooman et al., 2015; Hise et al., 2020).

The aim of the study was to examine whether the application of geochemical, chemical (fractionation) and biological (biotests, bioindicators) indicators will allow for a comprehensive assessment of ecological risks related to the mobility, ecotoxicity and bioavailability of trace elements, as well as to indicate their origin (sources) in the bottom sediment of the Rożnów reservoir.

## Material and methods

### Sample collection

The bottom sediments were collected from the Rożnów reservoir which is located on the Dunajec River in Małopolskie Province, southern Poland. The studies of Tarnawski et al. (2017), Baran et al. (2019b) and Szara et al. (2020a, 2020b) provide more details on the Rożnów reservoir and the place where it is located. The bottom sediments were sampled at 46 points in three zones: inlet zone (10 samples), middle zone (22 samples) and outlet zone in the immediate vicinity of the dam (14 samples). The top layer of bottom sediments (0–15 cm) was collected with the use of the Ekman sampler (Szara et al., 2020a). The samples, under laboratory conditions, were air-dried at room temperature, homogenized, 2-mm sieved and put into polyethylene containers. The *Anodonta anatina* mussels were sampled from the Rożnów reservoir as follows: 7 individuals from the inlet zone, 2 individuals from the middle zone and 2 individuals from the outlet zone. The mussels were transferred to the laboratory in an ice cool box immediately after collection. To remove gut content, the water in which mussels were kept for 24 h was filtered and the conditions in the container were identical to those in the natural environment. The samples were lyophilized and homogenized in a mortar.

### Chemical analysis

A four-step sequential chemical extraction scheme modified by BCR method was used for the quantitative determination of trace elements (As, Cd, Cr, Cu, Ni, Pb, Zn) in individual fractions of bottom sediments (BCR 2001, Baran et al., 2019a). The determination revealed the following four element fractions: exchangeable fraction (F1 – ion exchange and carbonate, CH_3_COOH with a concentration of 0.11 mol∙ dm^−3^ and pH = 2); reducible fraction related to hydrated Fe oxides and Mn oxides (F2 – reduction, extractable with NH_2_OHHCl with a concentration of 0.5 mol ∙ dm^−3^ and pH = 1.5); oxidizable fraction related to organic matter (F3 – oxidation, extractable with hot 30% H_2_O_2_ and further the mineralization products re-extracted with CH_3_COONH_4_ with a concentration of 0.5 mol ∙ dm^−3^ and pH = 2); and residual fraction, i.e., elements bound to minerals (F4 – hot digested in a mixture of HNO_3_ and HClO_3_ acids (3:2) v/v) (Baran et al., 2019a). The solutions of all fractions were centrifuged and filtered. The residues of all fractions were washed with distilled water and subjected to another centrifugation. The mussels were digested in a microwave (AntonPaar Multiwave 3000 microwave system) in a mixture of HNO_3_ and HCl (6:1 v/v) acids (suprapure, MERCK) to assess the trace element concentrations in them.

The element concentration in mussels and bottom sediments was determined with the use of an inductively coupled plasma optical emission spectrophotometer—Perkin Elmer ICP-OES Optima 7300 DV. The analytical method was assessed using the reference material BCR-701 (trace element fraction in sediments) and ERM – CE278k (trace elements in mussels). The results showed that the percentage of recovery was in the range between 79% Cr and 124% Pb (fractionation) as well as between 79% As and 96% Zn (total in mussels).

### Ecotoxicological analysis

Two biotests: Ostracodtoxkit and Microtox were used to evaluate the ecotoxicity of the bottom sediments (Baran et al., 2016; Szara et al., 2020b). The endpoint in the test was the inhibition of *Aliivibrio fischeri* luminescence. The Microtox biotest involved the measurement of the inhibition of bacterial luminescence after 15 min of samples exposure. A standard test procedure was used for sediment elutriate: 81.9% screening test. The analysis of changes in luminescence was performed using a Microtox M500 Analyzer (Microbics Corporation, 1992). The mortality and growth inhibition of the *Heterocypris incongruens* crustacean after a 6-day contact with the sediment was measured with the use of the Ostracodtoxkit biotest (Ostracodtoxkit, 2001; Cooman et al., 2015).

### Assessment of potential ecological risks

#### Chemical and geochemical indicators

The potential bioavailability factor (PBF) and the RAC (Risk Assessment Code) classification were applied to assess hazards related to the potential mobility of heavy metals in the bottom sediments (Al-Mur, 2020; Bielicka-Giełdoń et al., 2013; Klink et al., 2019; Singh et al., 2005). The RAC classification of metals bound to fraction I is as follows (in F1 [%]: < 1 no risk; 1–10 low risk; 11–30 medium risk; 31–50 high risk; 50 > very high risk) (Baran & Tarnawski, 2015; Singh et al., 2005). The PBF was calculated as the ratio of the potential mobile fraction (PMF∑F1-F3) of elements to the total element content (Bielicka-Giełdoń et al., 2013). The residual fraction (F4) was less mobile that the PMF ∑F1-F3 of elements (Aguilar-Hinojosa et al., 2016). The fraction 4 is related to the structure of minerals, and trace elements bound to it are stable and have no effect on water or organisms (Martinez-Santos et al., 2015; Baran et al., 2019a). The level of contamination and potential ecological risks related to trace elements in sediments were analyzed using single-element (Geoaccumulation Index, Contamination Factor, Potential Ecological Risk Factor) and multi-element (Pollution Load Index, Potential Ecological Risk Index) geochemical indicators (Table 1) (Kulbat & Sokołowska, 2019; Tytła & Kostecki, 2019). The geochemical background values by Bojakowska (2001): As – 5 mg; Cd – 0.5 mg; Cr and Ni – 5.0 mg; Cu – 6 mg; Pb – 10 mg; Zn – 48 mg ∙ kg^−1^ were adopted for calculations.

**Table 1:** The criteria of contamination level of toxic elements in bottom sediment

Indices*	Equation with description	Category	Category with description and abbreviations
Geoaccumulation Index (Igeo) of single element	${I}_{geo} = {log}_{2}\left(\frac{{C}_{n}}{\mathrm{1,5}{B}_{n}}\right){C}_{n}-$ measured concentration of element in the sediment sample, ${B}_{n}-$ geochemical background value in the Earth’s crust	*I*_geo_ ≤ 0	Practically uncontaminated (PUC)
		0 < *I*_geo_ ≤ 1	Uncontaminated to moderately contaminated (U-MC)
		1 < *I*_geo_ ≤ 2	Moderately contaminated (MC)
		2 < *I*_geo_ ≤ 3	Moderately to heavily contaminated (M-HC)
		3 < *I*_geo_ ≤ 4	Heavily contaminated (HC)
		4 < *I*_geo_ ≤ 5	Heavily to Extremely contaminated (H-EC)
		5 < *I*_geo_	Extremely contaminated (EC)
Contamination Factor (CF) index of single element (Hakanson 1980)	CF = $\frac{{C}_{0-1}^{i}}{{C}_{n}^{i}}$	CF < 1	Low contamination (LC)
	${C}_{0-1}^{i}$—mean content of elements from at list five sampling sites,	1 < CF < 3	Moderate contamination (MC)
	${C}_{n}^{i}$—concentration of elements in the Earth’s crust	3 < CF < 6	Considerable contamination (CC)
		CF > 6	Very high contamination (VHC)
Potential Ecological Risk Factor ($${E}_{r}^{i})$$ index of single element (Hakanson 1980)	$${E}_{r}^{i}= {T}_{r}^{i}\times {C}_{f}^{i}= {T}_{r}^{i}\times ({C}_{i}/{C}_{0}$$	$${E}_{r}^{i}$$< 40	Low risk (LR)
	${C}_{f}^{i}-$ contamination factor of the trace elements,	40 < ${E}_{r}^{i}$< 80	Moderate risk (MR)
	${C}_{i}-$ concentration of metal i in bottom sediment,	80 < ${E}_{r}^{i}$< 160	Considerable risk (CR)
	${C}_{0}-$ background value of the metal in the study area,	160 < ${E}_{r}^{i}$< 320	High risk (HR)
	${T}_{r}^{i}-$ biological toxicity factor of an individual element, which was determined for Zn = 1, Cr = 2, Cu = Pb = Ni = 5, As = 10, Cd = 30 (Hakanson 1980; Baran et al. 2016)	${E}_{r}^{i}$>320	Very high (VHR)
Pollution Load Index (PLI) of multi-element	PLI = (C_f1_ x C_f2_ x … x C_fn_)^1/n^	0 < PLI ≤ 1	Unpolluted (UP)
	*C*_f_—contamination factor for individual metals:	1 < PLI ≤ 2	Moderately to unpolluted (MUP)
	*C*_f_ = *C*_i_*/C*_0_,	2 < PLI ≤ 3	Moderately polluted (MP)
	*C*_i_—concentration of metal *i* in bottom sediment,	3 < PLI ≤ 4	Moderately to highly polluted (MHP)
	*C*_0_—background value of the metal in the study area,	4 < PLI ≤ 5	Highly polluted (HP)
	*n* = number of metals	PLI > 5	Very highly polluted (VHP)
Potential Ecological Risk Index (PERI) of multi-element (Hakanson 1980)	$\mathrm{PERI}=\sum_{i=1}^{n}{E}_{r}^{i}$	PERI < 150	Low risk (LR)
		150 < PERI < 300	Moderate risk (MR)
		300 < PERI < 600	Considerable risk (CR)
		PERI > 600	High risk (HR)

*Hakanson (1980), Kulbat and Sokołowska (2019), Tytła and Kostecki (2019)

#### Bioaccumulation factor and ecotoxicity assessment

The risk of accumulating trace elements from the sediments by the mussels was assessed by calculating the biota-sediment accumulation factor (BASF):

$$ BASF = \frac{Cm}{{Cs}} $$

where Cm—concentration of each metal in the mussels; Cs—concentration of each metal in the bottom sediments.

The BASF values classify organisms as: macroconcentrators (BASF > 2), microconcentrators (1 < BASF < 2) and de-concentrators (BASF < 1) (Szefer et al., 1999; Ziyaadini et al., 2017). The sediment toxicity was evaluated using the toxicity assessment method developed by Persoone et al. (2003): PE (percent toxic effect) < 20% no toxic effect; 20% ≤ PE < 50% low toxic sample; 50% ≤ PE < 100% toxic sample; PE—100% very toxic sample.

### Statistical analysis

The mean, standard deviation and the coefficient of variation (CV%) were calculated. The principal component analysis (PCA) and Pearson’s correlation matrix were applied to analyze the possible relationships between biological, chemical and ecotoxicological data. The Statistica 13 software was used for all statistical analyses.

## Results and discussion

### Fraction of trace elements in bottom sediments

**Fig. 1:**
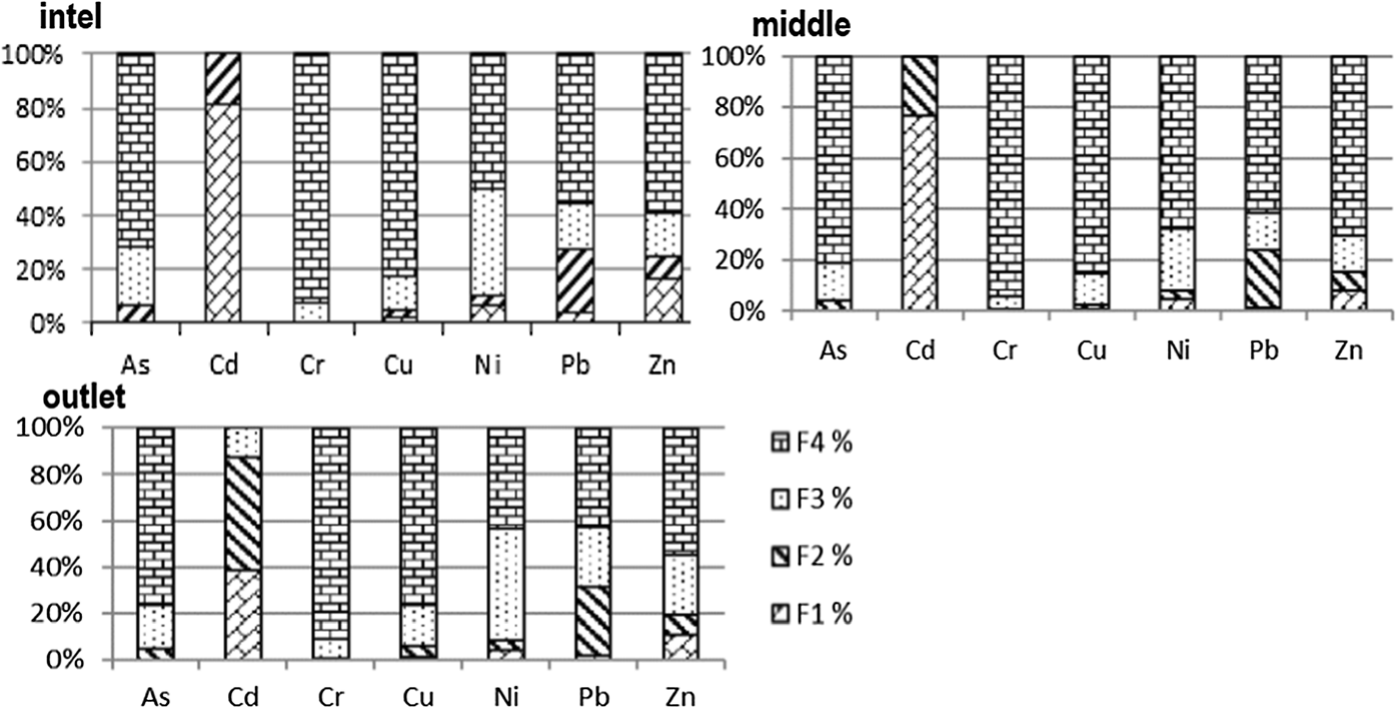
Fractional distribution and speciation of heavy metals in the bottom sediments

The mobility and potential toxicity of trace elements for living organisms depend on their presence in bioavailable chemical forms (Baran et al., 2019a; Morillo et al., 2008). The fractional analysis made it possible to distinguish individual fractions of trace elements (F1—exchangeable, F2—reducible F3—oxidizable, F4—residual) present in the tested sediment. The highest concentration of arsenic was found in the residual fraction (F4) 4.09 mg/kg (Table 2). For the reducible (F2) and oxidizable (F3) fractions, the average concentration of arsenic was 0.24 mg/kg and 0.89 mg/kg, respectively. In the exchangeable fraction (F1), the average concentration of the element was very low (0.003 mg/kg). Cadmium was mainly bound to exchangeable and reducible fractions (F1 = 0.14 mg/kg, F2 = 0.09 mg/kg). In the oxidizable fraction (F3), the cadmium concentration was on average 0.02 mg/kg ^1^. This element was not found in the residual fraction (F4). Chromium was found primarily in the F4 fraction (58.96 mg/kg on average). In other fractions, the chromium content was lower, F3 (4.06 mg/kg), F2 (0.32 mg/kg) and F1 (0.18 mg/kg). The distribution of copper, nickel and zinc concentrations in individual fractions was as follows: The highest concentrations were found in the residual fraction (23.36 mg/kg Cu, 21.44 mg/kg Ni, 44.69 mg/kg Zn on average), followed by the oxidizable fraction (3.99 mg/kg Cu, 12.80 mg/kg Ni, 12.76 mg/kg Zn on average) and the lowest in exchangeable (0.83 mg/kg Cu, 1.79 mg/kg Ni, 7.26 mg/kg Zn on average) and reducible (0.78 mg/kg Cu, 1.42 mg/kg Ni, 5.22 mg/kg Zn on average) fractions. Lead was mainly bound to the residual fraction (6.09 mg/kg on average). In other fractions, the lead concentration was on average 0.21 mg/kg (F1), 2.79 mg/kg (F2), 2.10 mg/kg (F3). In summary, the trace element content in the tested sediment was found in the individual fractions in the following order: As and Cr: residual (F4) > oxidizable (F3) > reducible (F2) > exchangeable (F1); Cd: exchangeable (F1) > reducible (F2) > oxidizable (F3) > residual (F4); Cu, Ni, Zn: residual (F4) > oxidizable (F3) > exchangeable (F1) > reducible (F2); Pb: residual (F4) > reducible (F2) > oxidizable (F3) > exchangeable (F1). The share of trace elements in individual fractions changed depending on the tested reservoir zone (Fig. 1). In the inlet, middle and outlet zones of the reservoir, the residual fraction (F4) dominated for all elements except for cadmium, representing 71–80% As; 91–94% Cr; 75–84% Cu; 43–68% Ni; 42–62% Pb; and 55–71% Zn, respectively. In the reservoir inlet zone, the share of elements in F1 was: 80% Cd; 17% Zn; 7% Ni; 4% Pb; 2% Cu; < 1% Cr. No arsenic was found in the exchangeable fraction. Cadmium was only bound to the exchangeable fraction (F1) and the reducible fraction (F2). In the reservoir middle zone, on average < 1% As and Cr; 73% Cd; 1% Cu, Pb; 5% Ni; 8% Zn in the exchangeable fraction (F1) were found. In this zone, cadmium was also present only in exchangeable (F1) and reducible (F2) fractions. The percentage of elements in the F1 fraction in the reservoir outlet zone was as follows: 1% As, Cr; 43% Cd; 1% Cu; 4% Ni; 2% Pb; 11% Zn.

**Table 2:** Content of trace elements (mean ± SD) in bottom sediments (*n* = 46) and mussels (*n* = 10)

Materials	As	Cd	Cr	Cu	Ni	Pb	Zn	
	Content of trace elements in different fractions mg/kg							
Bottom sediment	F1	0.003 ± 0.01	0.14 ± 0.04	0.18 ± 0.05	0.83 ± 0.29	1.79 ± 0.51	0.21 ± 0.10	7.26 ± 3.74
	F2	0.24 ± 0.11	0.09 ± 0.21	0.32 ± 0.08	0.78 ± 0.62	1.42 ± 0.37	2.79 ± 0.76	5.22 ± 1.07
	F3	0.89 ± 0.19	0.02 ± 0.04	4.06 ± 1.44	3.99 ± 4.30	12.80 ± 5.26	2.10 ± 1.06	12.76 ± 5.22
	F4	4.09 ± 1.01	0.00	58.96 ± 11.82	23.36 ± 14.79	21.44 ± 7.67	6.09 ± 1.46	44.69 ± 11.93
	Total*	5.24 ± 1.11	0.26 ± 0.13	63.23 ± 12.66	28.65 ± 18.81	37.11 ± 7.69	11.15 ± 1.95	69.69 ± 12.73
	PBF	0.23 ± 0.07	1.00 ± 0.02	0.07 ± 0.02	0.21 ± 0.16	2.56 ± 1.00	0.45 ± 0.11	0.37 ± 0.11
	Risk**	% of samples						
	No	100	0	97	3	3	17	0
	Low	0	4	3	91	89	80	67
	Medium	0	3	0	5	8	3	26
	High	0	17	0	0	0	0	7
	V. high	0	75	0	0	0	0	0
Mussels	Content mg/kg	As	Cd	Cr	Cu	Ni	Pb	Zn
		5.66 ± 1.79	0.68 ± 0.51	2.11 ± 0.97	6.80 ± 3.21	1.04 ± 0.38	0.73 ± 0.28	141.1 ± 56.3
		*2.93–10.96*	*0.15–1.82*	*0.56–4.43*	*3.47–16.25*	*0.42–1.95*	*0.20–1.27*	*55.5–306*
	BASF	1.08 ± 0.34	2.63 ± 1.96	0.03 ± 0.02	0.24 ± 0.11	0.03 ± 0.01	0.07 ± 0.03	2.02 ± 0.81
		*0.56–2.04*	*0.57–7.00*	*0.01–0.07*	*0.12–0.57*	*0.01–0.05*	*0.02–0.11*	*0.80–4.40*

*Szara et al. (2020a), *Elements in mobile fraction MF [%]: < 1 no risk; 1–10 low risk; 11–30 medium risk; 31–50 high risk; 50 > very high risk (Singh et al., 2005). *PBF *potential bioavailability factor; *BASF* biota-sediment accumulation factor; values in italic mean the range from minimum to maximum

Generally, trace elements in the exchangeable fraction (F1) are considered more mobile and bioavailable. Based on the RAC classification, which takes into account the presence of trace elements in the exchangeable fraction (F1), it was found (Table 2): no risk related to the release of arsenic (100% of samples) and chromium (97% of samples) into the environment, low risk related to the release of copper (91% of samples), nickel (89% of samples), lead (80% of samples) and zinc (67% of samples) and very high risk of cadmium release (75% of samples). The highest share of potential mobile fraction (PMF ∑F1-F3) was found for Cd (92%); Ni, Pb (47%); Zn (39%); As (24%), Cu (18%) and Cr (8%) of the mean total element content. The presence of trace elements in potential mobile forms (PMF ∑F1-F3) probably indicates their anthropogenic source, while in the fourth fraction (F4), elements of lithogenic origin are usually available (Baran et al., 2019a). It was observed in the study that the percentages of trace elements bound in the PMF (without Cd) were from 1.2 (Ni, Pb) to 12 (Cr) fold lower than those of the F4. This implies that Cr, Cu, As, Ni, Pb and Zn generally came from natural and anthropogenic inputs and Cd and form anthropogenic sources. The residual fraction (F4) of trace elements represented on average 76% As, 0% Cd, 93% Cr, 80% Cu, 54% Ni, Pb and 61% Zn of their total content. The potential bioavailability factor (PBF) achieved the following mean values 2.56 Ni > 1.00 Cd > 0.45 Pb > 0.37 Zn > 0.23 As > 0.21 Cu > 0.07 Cr (Table 2). The high PBF values for nickel and cadmium were probably due to their potential toxicity, as they are easily released into the aquatic environment, ingested by organisms and entered into the food chain in studied reservoir.

Other researchers found that the trace elements bound to the F1 fraction, in addition to being the most mobile and bioavailable, also provide information on the recent bottom sediment contamination (Klink et al., 2019). In our study, Cd had the highest percentage bound to the F1 fraction of all trace elements. The presence of a trace element in fractions F2 (reducible) or F3 (oxidizable) may be related to its emission from anthropogenic sources in an earlier period (Klink et al., 2019). Additionally, the mobility and bioavailability of elements in F2 and F3 are strongly dependent on physical and chemical factors (availability of oxygen, redox potential, pH, microbial activity). In the present study, Ni, Zn, Cu, Pb (F3) and Pb (F2, F3) are metals whose percentage was a bit high in reducible (F2) and oxidizable (F3) fractions (Fig. 1). In the study by Copaja et al. (2014) on the metal content in individual fractions in the bottom sediments of the river Choapa, the authors found that Cr and Ni were present mainly in the reducible (F2) and residual (F4) fractions, the concentration of Cu and Zn was similar in all fractions, while Pb was found mainly in the reducible (F2) fraction. On the other hand, Zhangb et al. (2019) demonstrated the dominance of the residual fraction (F4) for Zn, Pb and Cu in Dredged Marine Sediments from Bohai Bay, China. In the study of Baran and Tarnawski (2013) concerning bottom sediments of similar granulometric composition, i.e., with a high content of silt and clay fractions, the highest mobility was demonstrated for Zn, followed by Pb ≈ Ni > Cu. The largest concentrations of metals were bound to the F4 fraction, while the lowest were found in the forms most easily accessible to living organisms in the F1 fraction. In turn, high organic matter content in the bottom sediments influenced the share of metals in particular fractions more than other physical and chemical parameters of these sediments (Baran et al., 2019a; Baran, & Tarnawski, 2015). Moreover, among the studied fractions, the highest metal concentration was bound to the organic fraction F3, which indicates that this is the most dominant metal form in the sediment of the Rybnik reservoir (Baran & Tarnawski, 2015). The higher content of organic matter in the sediments also increased the share of trace elements in the potential mobile fraction (PMF ∑F1-F3) and, at the same time, decreased the binding of elements in the mobile fractions (F1) (Baran et al., 2019a).

### Assessment of contamination level of trace elements in bottom sediments

Many authors consider that the key factor for using geochemical parameters is to find the difference between trace elements of anthropogenic origin and those from natural sources (Birch, 2017). The Geoaccumulation Index (Igeo) values showed that, depending on the tested element, the bottom sediments were practically uncontaminated (As, Cd, Pb, Zn), moderately contaminated (Cu), moderately to heavily contaminated (Ni), heavily contaminated (Cr) (Table 3). Igeo had the following order: Cr > Ni > Cu > Zn > Pb > As > Cd. The second tested single-element indicator was the Contamination Factor (CF) on the basis of which the elements formed the same series as in the case of Igeo: Cr > Ni > Cu > Zn > Pb > As > Cd. The sediments were characterized by low contamination (Cd), moderate contamination (As, Pb, Zn), considerable contamination (Cu), and very high contamination (Cr, Ni). The Potential Ecological Risk Factor (E^i^_r_) showed that the bottom sediments were characterized by a low contamination risk related to the content of all trace elements (E^i^_r_: As = 10,44; Cd = 15,58; Cr = 25,23; Cu = 23,87; Ni = 37,11; Pb = 5,58; Zn = 1,45). Another group of indicators were multi-element indicators, calculated for 7 tested elements. The Pollution Load Index (PLI) value for the studied sediment was 2.10, which indicates moderate sediment contamination. The last analyzed indicator was the Potential Ecological Risk Index (PERI) whose value indicated a moderate risk (PERI = 119.23) (Table 3). In our previous studies on the analysis of core bottom sediment samples from the Rożnów reservoir, similar relationships were shown, i.e., Cr and Ni were found to be significant contaminants of the Rożnów reservoir bottom sediments (Baran et al., 2019b). Also in the study by Szara et al (2020a), using consensus-based sediment quality guideline (the TEC and PEC values), it was demonstrated that 2% of samples were above the PEC values for Ni and the TEC values were exceeded for Ni and Cr in 93% of samples. The mean PECq of trace elements was 0.26, meaning that the total trace element content was likely to cause moderate potential toxicity to the biological communities in the bottom sediments (Szara et al., 2020a).

**Table 3:** Assessment of sediment contamination level and potential ecological risk (*n* = 46)

Metal	Igeo**		CF		${E}_{r}^{i}$		PLI		PERI	
As	−0.52	PUC*	1.04	MC	10.44	LR	2.10	MP	119.23	LR
Cd	−1.53	PUC	0.52	LC	15.58	LR				
Cr	3.07	HC	12.62	VHC	25.23	LR				
Cu	1.67	MC	4.77	CC	23.87	LR				
Ni	2.31	M-HC	7.42	VHC	37.11	LR				
Pb	−0.43	PUC	1.12	MC	5.58	LR				
Zn	−0.05	PUC	1.45	MC	1.45	LR				

*PUC Practically uncontaminated, *HC* heavily contaminated, *MC* moderately contaminated, *M-HC* moderately to heavily contaminated, *LC* low contamination, *VHC* very high contamination, *CC* considerable contamination, *LR* low risk, *MP* moderately polluted, **Igeo Geoaccumulation Index, *CF* Contamination Factor, *E*^*i*^_r_ Potential Ecological Risk Factor, *PLI* Pollution Load Index, *PERI* Potential Ecological Risk Index

### Bioconcentration of trace elements in mussels

The trace element concentration in the mussel tissues varies from 2.93 to 10.96 mg As, 0.15 to 1.82 mg Cd, 0.56 to 4.43 mg Cr, 3.47 to 16.25 Cu, 0.42 to 1.95 mg Ni, 0.20 to 1.27 mg Pb and 55.5 to 306 mg/kg Zn (Table 2). Our study results showed the following overall pattern of trace elements in the mussels: Zn > Cu > As > Cr > Ni > Pb > Cd. The highest contents of arsenic (6.55 mg/kg), cadmium (1.64 mg/kg), chromium (3.22 mg/kg) and nickel (1.09 mg/kg) were determined in mussels collected in the outlet zone of the reservoir. Mussels from the inlet zone had the highest copper content (7.59 mg/kg). In the case of lead, the mussels of each zone contained similar levels of the element. In turn, the highest zinc contents were recorded for mussels collected in the reservoir middle zone. The BASF calculated for the mussels of the Rożnów reservoir ranged from 0.01 (Cr) to 7.00 (Cd) (Table 2). The highest mean values of BASF were found for Cd (2.63), followed by Zn (2.02) > As (1.08) > Cu (0.24) > Pb (0.07) > Cr, Ni (0.03). Considering all trace metals in the mussels collected in different zones of the reservoir, the highest BASF value was found in the outlet zone (As, Cd, Cr, Ni), middle zone (Pb, Zn) and inlet zone (Cu). Among the trace metals analyzed in mussels from all sampling locations in the present study, the concentration and the BASF value varied significantly only for Cd. Generally, given the BASF values, it can be concluded that mussels had a more active role in the accumulation and binding of Cd and Zn (macroconcentrator) and lower role for As (microconcentrator). It is also notable that, at all stations, mussels showed very low BASF for Cr, Ni, Pb, and Cu (deconcentrator). According to Ziyaadini et al. (2017), macroconcentrators can be particularly suitable biomonitors to determine the relationship between the concentration of a given metal in the organism and its bioavailability for bottom sediments. Therefore, *A anatina* can be used specifically for the measurement of cadmium in the aquatic environments.

### Ecotoxicity of bottom sediments

**Fig. 2:**
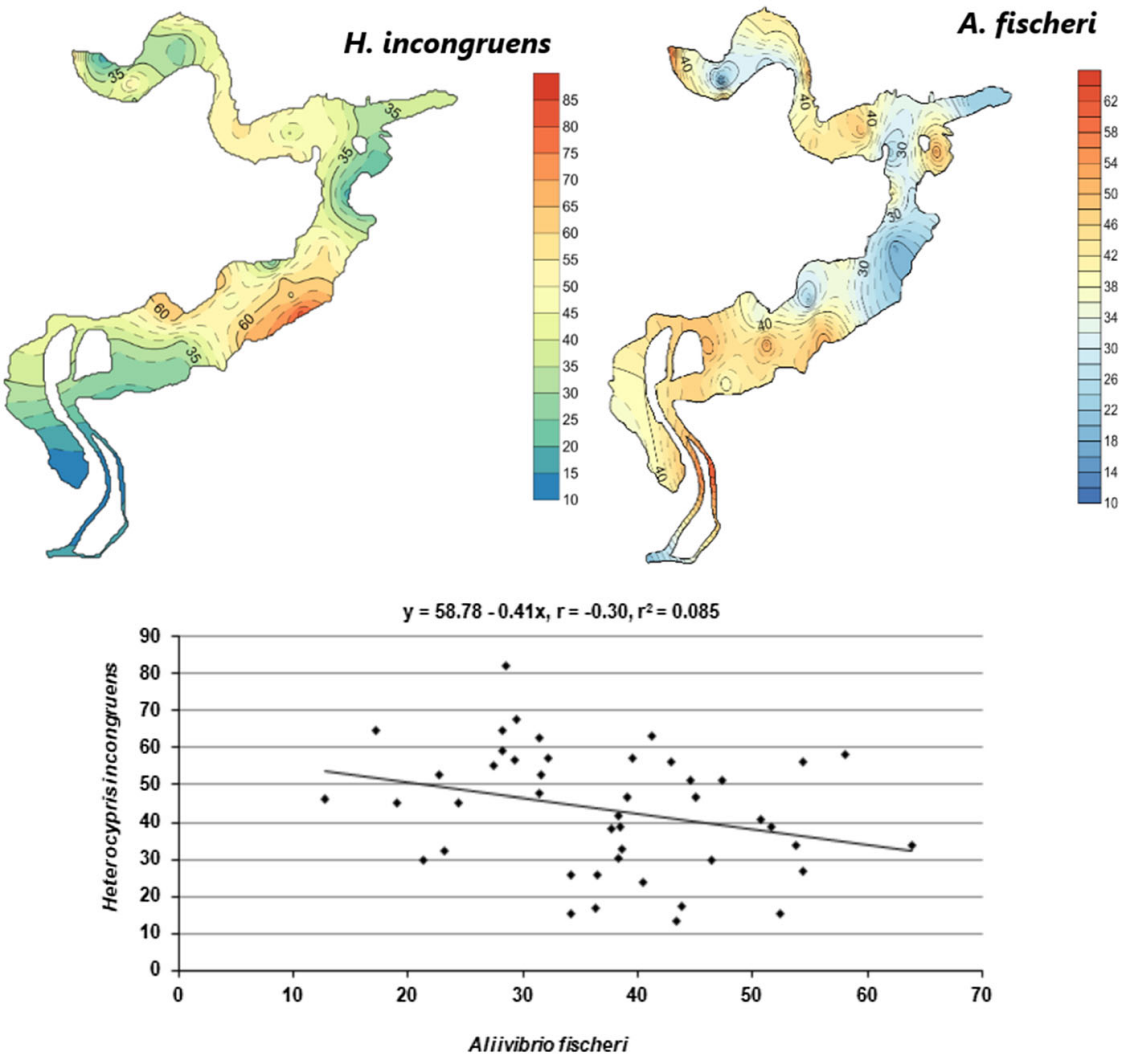
Spatial distribution of luminescence inhibition (PE%) of A. *fischeri* and growth inhibition (PE%) of *H. incongruens* in the sediments (*n* = 46) and data pair correlations of the both response of tested organisms

The ecotoxicity of sediments was characterized by a low spatial variability, CV = 40% (inhibition of *H. incongruens* growth) and CV = 31% (inhibition of *A. fischeri* luminescence) (Fig. 2). The bottom sediments were more toxic to *Heterocypris incongruens* than *Aliivibrio fischeri* (Fig. 2). The *H. incongruens* growth inhibition was 11–100%, with an average of 43%, while the mortality rate ranged from 0 to 100%, with an average of 10%. The highest inhibition of crustacean growth was found in the bottom sediment sampled from the middle zone (PE 47%) and the lowest in the inlet zone (PE 34%). In turn, the highest mortality of *H. incongruens* was observed in the reservoir inlet zone (PE 17%) and the lowest in the outlet zone (3%). The mean percent inhibition of *A. fischeri* luminescence was 37% and ranged from 13 to 64%. In the middle zone of the reservoir, the luminescence inhibition was the lowest (PE 34%), while its highest value was observed in the inlet zone (PE 43%).

Most of the tested sediment samples had low toxicity in relation to the tested organisms. For *H. incongruens*, 11% of the samples were non-toxic, 50% of the samples had low toxicity, and 39% of the samples were toxic. For *A. fischeri*, no toxicity was found in 7% of the samples, low toxicity in 76% of the samples and toxicity in 17% of the sediment samples.

A negative correlation was found between luminescence inhibition in *A. fischeri* and growth inhibition in *H. incongruens* (r =—0.30 p < 0.05), meaning differences in the sensitivity of tested organisms to toxicants present in the sediments (Fig. 2). However, the analysis indicated that the correlation between the organism was of low statistical significance and explained only 8% of the variability (Fig. 2).

## Correlation and PAC analyses

The correlation between the contaminant content in the environment and in organisms is a useful method to evaluate the monitoring potential of organisms (Klink et al., 2019; Vignati et al., 2019). Numerous data indicate that there are direct relationships between the element content in the benthic organism and bottom sediments (Shirneshan et al., 2013). Table 4 shows correlations between different geochemical fractions of trace elements in the sediment and the mussel tissues. Cadmium in the tissues was strongly and very strongly significantly positively correlated with all analyzed metal fractions (r = 0.715 F1, F4 – 0.968 F3, *p* < 0.05). Significant, strong and very strong correlations were also determined between As, Zn in the tissue and the content of both elements in F1, F4, and the total content of Zn and As in the sediment. In addition, the study revealed negative significant correlations between the total content, F4 and F2 of Cr in the sediment and the element content in the tissue of *A. anatine* (Table 4). Fractions 1 and 2 of Cu were significantly correlated with the Cu content in the mussels. For Ni (F4, total) and Pb (F2, F3), there were significant negative relationships between their content in sediment and in the tissue (Table 4). The correlation analysis results confirmed our data relating to the BASF factor (Table 2). The analysis revealed high (Cd, Zn) and moderate (As) accumulation in mussel tissues, as well as the most significant positive correlations for these elements (Table 4).

**Table 4:** Correlation coefficients of trace element fractions in sediments and their total content in mussels

Sediment *n* = 10	Mussels *n* = 10						
	As	Cd	Cr	Cu	Ni	Pb	Zn
Total	0.996*	0.907*	−0.981*	−0.225	−0.808*	−0.583	0.967*
F1	0.706*	0.715*	−0.112	0.899*	0.597	0.249	−0.970*
F2	−0.485	0.943*	−0.730*	0.904*	−0.312	−0.950*	0.628*
F3	0.190	0.968*	−0.476	0.436	0.260	−0.996*	0.122
F4	0.961*	0.715*	−0.998*	−0.447	−0.957*	0.444	0.954*

*Bold values represent correlation coefficients significant at the level of *p* < 0.05: 0 < r < 0.3 very low correlation; 0.3 ≤ r < 0.5 low correlation; 0.5 ≤ r < 0.7 medium correlation; 0.7 ≤ r < 0.9 strong correlation;0.9 ≤ r < 1 very strong correlation

The total content of trace elements in sediments had no effect on their bioavailability and mobility in water ecosystems; however, their chemical forms had ecotoxicological value (Shirneshan et al., 2013). On the other hand, the authors of the present study believe that the problem of mobility and bioavailability of trace elements is more complex and can be controlled both directly, by the total trace element content, and indirectly, related to the presence of the element in a given fraction; it can also depend on the properties of the element itself and the physicochemical factors of the medium. In our study, cadmium deserves particular attention, as a significant positive correlation was found between the Cd content (total, all fractions) in the sediments and its content in mussel tissues, which confirmed that cadmium was highly mobile in the environment shown in the fractional analysis. In turn, the total Cd content in the bottom sediments was generally low and the geochemical indicators showed no significant contamination of the bottom sediments with cadmium (Table 3). Therefore, when assessing the bioavailability or the degree of bioaccumulation of a given element in bottom sediments, one should take into account their total content as not to overestimate their risk to living organisms.

However, it should be noted that fractional analysis is a chemical process and knowledge of the relationship between the element fraction in the bottom sediments and element bioaccumulation in aquatic organisms provides important information on potential, rather than actual, interaction with biotic components of the aquatic environment. The correlation between the presence of metal in F1 (ion exchange fraction) and its content in living organisms gives better information on its bioavailability and can be used to predict the bioaccumulation of trace elements, as the element presence in this fraction is considered a potential hazard to aquatic organisms (Singh et al., 2005; Shrineshan et al., 2013; Baran & Tarnawski, 2015, Klink et al., 2019). In our study, the As, Cd, Cu contents in F1 correlated significantly positively with the content of these metals in mussel tissues. There was no positive correlation between zinc F1 and its content in bivalve mussel tissues. Shirnshan et al. (2013) obtained similar results. Attention should also be paid to the positive, although insignificant, statistical correlation between the Ni content in the ion exchange fraction (F1) and its content in mussel tissues. For nickel, the study revealed the highest value of the potential bioaccumulation factor (PBF) estimated on the basis of fractional analysis and the lowest value of the bioaccumulation factor (BASF) calculated on the basis of the metal content in mussel tissues and bottom sediments (Table 2). It should be highlighted that both nickel and cadmium and their compounds are on the list of priority hazardous substances for water quality. What is more, they should be completely eliminated from the aquatic environment due to their highly toxic properties, bioaccumulation susceptibility and persistence (Directive 2013/39/EU of the European Parliament and of the Council of 12 August 2013). Very interesting correlation relationships were also found for the Cr content in individual fractions and in mussel tissues. These relationships were mostly significantly negatively correlated. As shown by geochemical indicators (Table 3) as well as our previous studies, chromium contaminates bottom sediments of the Rożnów reservoir (Baran et al., 2019b; Szara et al., 2020a). Studies by other authors have also shown that the chromium content in the bottom sediments of the Dunajec River, which feeds the two dam reservoirs: Rożnów and Czorsztyn, is also increased (Szalińska et al., 2013; Vignati et al., 2019). However, the bioaccumulation factor (BASF) calculated in this study for Cr was low, as was the potential bioaccumulation factor (PBF) (Table 2). In the study of Vignati et al. (2019), the Cr bioaccumulation factor value for the *Chironomus* sp. in the bottom sediment of the Czorsztyn reservoir (compensating reservoir for the Rożnów reservoir) was higher and ranged from 0.1 to 1.3 (Vignati et al., 2019). The higher bioaccumulation factor value may be due to the fact that *Chironomus* sp. are organisms that feed on the bottom sediment particles, while mussels are filter feeders and indicate the mobility of a given element from the sediment to supersedimentary water. Our study has also shown that, under the physicochemical conditions in the bottom sediments of the Rożnów reservoir, the mobility of chromium is very low despite the inflow of Cr with tannery wastewater (Table 2, Fig. 1). It may also indicate that the main form of chromium entering the tanning sludge is the reduced form of Cr (III), possibly with limited bioavailability and low ecotoxicity (Vignati et al., 2019).

Table 5 shows results of the analysis of correlation between the trace element content in individual fractions and the test organism response. The most statistically significant positive correlations were found between the content of trace elements As, Cr, Ni, Pb, Zn (total content, F4) and *H. incongruens* growth inhibition. In case of *H. incongruens* growth inhibition, significant relationships with Cd (F1, positive relationship) and Pb (F1, negative relationship) were also found. The mortality of *H. incongruens* was generally negatively correlated with the content of elements in individual fractions, and statistically significant relationships were discovered only for Cr (F1, F2, F3) and Ni, Pb, Zn (F3). For *A. fischeri*, significantly positive relationships were observed with metals present in F1 (Cu), F2 (Cu, Pb) and F3 (Cd, Cr, Ni, Pb) as well as negative relationships with F1 (Pb). It should be noted that, for all relevant relationships, the obtained Person’s correlation coefficients were low or very low (Table 5). This can be explained by several factors. First of all, as stated in Sect. 4, most sediment samples showed relatively low toxicity to the test organisms and, as stated in Sect. 3.1, they were barely mobile. Secondly, the lack of significant relationships between the trace element content in individual fractions and the response of test organisms suggests their relatively low mobility and bioavailability (except for Cd). Additionally, other contaminants such as ammonia, pesticide residues, PAHs, biogens or dioxins may be present in the Rożnów reservoir sediments, having effect on the trace element toxicity (Baran et al., 2019b; Szara et al., 2020a). Furthermore, the interactions between different substances present in the sediment may cause synergistic or antagonistic effects that are difficult to predict and analyze (Apitz, 2011; Baran et al., 2016; de Castro-Catala et al., 2016; Hise et al., 2020). The correlation analysis showed that *A. fischeri* was a less sensitive organism than *H. incongruens*. What is more, the crustacean growth inhibition was associated with the content of trace element in stable forms—while the bacteria luminescence inhibition—with potentially mobile metal fractions (F2 and F3). The differences in the sensitivity of the tested organisms concern not only the species, trophic group or contamination type, but also the way they are exposed to toxic substances. In the Ostracodtoxkit test, crustaceans were exposed to both soluble contaminants and contaminants absorbed on sediment particles, while exposure of bacteria (Microtox) was limited to dissolved and thus more mobile substances. It is worth mentioning here that potentially mobile element fractions F2 and F3 positively correlated with inhibition of bacterial luminescence (Table 5). Our previous studies also showed a lower toxicity of the Rożnów reservoir bottom sediments to the test plants (*Lepidium sativum, Sinapis alba, Sorghum saccharatum*) than to *Thamnocephalus platyurus* (Szara et al., 2020a). On the other hand, Baran et al. (2016) evaluated the sensitivity of the performed tests and obtained a higher number of toxic responses for *A. fischeri* than for *H. incongruens* in sediments whose organic matter content was high.

**Table 5:** Correlation coefficients of trace element fractions and ecotoxicity of sediments

Parameters *n* = 46	Mortality of *H. incongruens*	Growth inhibition of *H. incongruens*	Inhibition of luminescence *A. fischeri*
Total As	−0.184	**0.333**	0.188
Total Cd	−0.093	0.179	0.251
Total Cr	−0.106	**0.345**	0.112
Total Cu	−0.005	0.189	−0.001
Total Ni	−0.041	**0.323**	0.073
Total Pb	−0.099	**0.368**	0.111
Total Zn	0.111	**0.471**	−0.031
F1 As	−0.170	0.082	0.162
F1 Cd	0.061	**0.311**	−0.198
F1 Cr	−**0.311**	−0.038	−0.039
F1 Cu	−0.265	0.016	**0.296**
F1 Ni	0.142	0.138	−**0.373**
F1 Pb	−0.113	−**0.442**	0.116
F1 Zn	0.125	0.146	0.060
F2 As	0.004	0.218	0.192
F2 Cd	−0.086	0.061	0.265
F2 Cr	−**0.352**	−0.018	0.285
F2 Cu	−0.235	−0.127	**0.343**
F2 Ni	−0.244	0.090	0.260
F2 Pb	−0.220	0.123	**0.295**
F2 Zn	−0.085	0.244	0.190
F3 As	−0.090	−0.035	0.133
F3 Cd	−0.162	−0.019	**0.344**
F3 Cr	−**0.379**	−0.024	**0.323**
F3 Cu	−0.148	0.041	0.145
F3 Ni	−**0.465**	−0.287	**0.391**
F3 Pb	−**0.346**	0.005	**0.372**
F3 Zn	−**0.305**	0.005	0.216
F4 As	−0.170	**0.355**	0.158
F4 Cr	−0.067	**0.370**	0.079
F4 Cu	0.055	0.178	−0.063
F4 Ni	0.272	**0.482**	−0.167
F4 Pb	0.245	**0.462**	−0.281
F4 Zn	0.222	**0.443**	−0.164

*Bold values represent correlation coefficients significant at the level of *p* < 0.05**:** 0 < r < 0.3 very low correlation; 0.3 ≤ r < 0.5 low correlation; 0.5 ≤ r < 0.7 medium correlation; 0.7 ≤ r < 0.9 strong correlation;0.9 ≤ r < 1 very strong correlation

Numerous factors control the chemical fractions of trace elements in bottom sediments (Baran & Tarnawski, 2015; Martinez-Santos et al., 2015; Aguilar – Hinojosa et al., 2016; Baran et al., 2019a; Klink et al., 2019). In our previous study, the analysis involved the pH, particle size, organic carbon and Fe contents (Szara et al., 2020a). The PCA extracted four positive correlations responsible for 72.55% of the dataset total variance (Table 6). The PC1, explaining 36.85% of the total variance, had strongly positive loadings (> 0.70) on silt, Fe, and stable forms of trace elements (total content and F4 of As, Cd, Cr, Ni, Pb, Zn) and negative loadings on sand. The second factor (PC2) explained 20.61% of the total variance and was dominated by clay and the trace element content in F2 (Cr, Ni, Pb) and F3 (As, Cr, Ni, Pb, Zn). The third factor was responsible for 9.05% of the total variance and was highly loaded on the content of Cd (total, F2, F3), As, Zn and Pb (F1) and Cu (F2) (Table 6). The fourth factor (PC4), i.e., only 6.04% of variance, displayed high loading values of F1 Cd, Cr, and Ni. It is believed that the total elemental content in sediments does not provide reliable information on anthropogenic sources, as it is strongly related to the properties of the bottom sediments (Klink et al., 2019). The results of the PCA partially confirmed the dependence indicated above. In our study, PC1 represented natural factors and sources determining the trace element content in the Rożnów reservoir bottom sediments. PC1 indicated that the silt fraction, which is dominant for the bottom sediment, has a significant effect on the total (As, Cd, Ni, Pb, Zn, Cr) and F4 (As, Cr, Ni, Pb, Zn) content of trace elements. A significantly positive correlation (PC1) was also demonstrated between the content of Fe and the content of most trace elements (total content, F4), which may also suggest that their sources were natural. PCA results also indicated significantly positive correlations between trace elements (total content, F4) in the Rożnów reservoir bottom sediments, which was related to similar sources and distribution routes, rather of natural origin. Hu et al. (2013) and Baran et al. (2019b) obtained similar results. The study revealed an insignificant relationship between the total element contents and fractions and the TOC contents in the sediments. As demonstrated in our previous studies, the reservoir rheolimnetic nature and the relatively low TOC content suggest that TOC is not the main factor affecting the content of trace elements and their distribution in individual fractions in the studied bottom sediments (Baran et al., 2019b). No significant effect of pH on the content and distribution of trace elements in bottom sediments was determined in the present study. The mobility of trace elements in the Rożnów reservoir sediments may be limited due to the neutral and alkaline sediment pH, as evidenced by the low element contents in the F1 and F2 fractions. The characteristics of trace elements bound to PC2 and PC3 indicate mixed (natural and anthropogenic) sources/control factors in the Rożnów reservoir sediments. As stated in Sect. 3.1, elements present in the F2 and F3 fractions are potentially mobile and their distribution may be related both to emissions from earlier anthropogenic sources and natural factors of the aquatic environment. We observed (PC2) a significant effect of the clay fraction on the element presence in the F2 and F3 fractions (Cr, Ni, Pb, Ni), and for other elements, this effect was also positive but statistically insignificant. At this point, it is worth mentioning that the Rożnów reservoir is the fastest silting dam reservoir in Poland (Szarek-Gwiazda, 2014; Baran et al., 2019b; Szara et al., 2020a, 2020b). The average amount of retained mineral material ranges between 470 thousand m^3^/year and 2150 thousand m^3^ / year and is the highest in Poland (Baran et al., 2019a). Silting is responsible for the inflow of small fractions whose origin is both natural and anthropogenic. The fine fraction, especially the clay fraction, has a large sorption area compared to trace elements. Geological structure of the catchment area, frequent floods, surface waters from cropland is the main factors affecting silting. Additionally, the main anthropogenic source here is the inflow of municipal sewage directly into the reservoir (Szara et al., 2020a; Szarek-Gwiazda, 2014). Iron and manganese geometry and thus the redox potential are the natural processes that significantly influence the behavior, form and the further activity of trace elements in the aquatic environment (Wang et al., 2012; Zhou, 2020). The results of a low redox potential are the reduction of sulfates as well as the formation of insoluble sulfides. Under reducing conditions, the formation of insoluble sulfides causes the immobilization and binding of some metals, such as copper, zinc, lead, cadmium, nickel, into immobile sulfides inaccessible to living organisms. On the other hand, in the case of manganese and iron, the lower the redox potential, the stronger the reduction processes and the hydrated manganese (IV) and iron (III) oxides in the sediment are reduced. Under anaerobic (reducible) conditions, trace elements absorbed on the developed surface of the precipitating hydrated iron and manganese oxides (F2) may be released from the sediment into the water. The PC2 showed that the content of elements in the F2 fraction positively correlated with the iron content; however, these correlations were statistically insignificant.

**Table 6:** PCA applied to the results of trace element fractions and chemical, physical and ecotoxicological properties of sediments

Parameters	PC1	PC2	PC3	PC4
Sand	−**0.918**	−0.211	−0.008	−0.059
Silt	**0.790**	−0.190	−0.137	0.018
Clay	0.374	**0.728**	0.253	0.081
TOC	0.238	−0.489	−0.062	−0.261
pH	0.098	0.010	−0.409	0.073
Fe	**0.731**	0.315	0.076	−0.038
Total As	**0.803**	0.468	0.135	−0.027
Total Cd	0.300	0.259	**0.861**	−0.117
Total Cr	**0.870**	0.445	0.105	0.105
Total Cu	0.510	0.039	0.048	0.119
Total Ni	**0.871**	0.360	0.148	0.159
Total Pb	**0.866**	0.432	0.126	0.003
Total Zn	**0.960**	0.151	0.047	−0.015
F1 As	0.363	−0.031	**0.793**	0.056
F1 Cd	0.021	0.484	0.083	**0.778**
F1 Cr	−0.132	0.358	−0.056	**0.803**
F1 Cu	0.162	−0.278	0.500	0.489
F1 Ni	−0.597	0.154	−0.286	**0.797**
F1 Pb	0.014	−0.275	−0.094	0.062
F1 Zn	0.048	0.119	**0.796**	0.123
F2 As	0.356	0.195	0.266	0.163
F2 Cd	0.070	0.120	**0.959**	−0.158
F2 Cr	0.240	**0.788**	0.369	0.200
F2 Cu	−0.083	0.433	**0.855**	−0.032
F2 Ni	0.502	**0.707**	0.265	0.200
F2 Pb	0.398	**0.650**	0.572	0.144
F2 Zn	**0.**549	0.462	0.265	0.065
F3 As	−0.007	**0.698**	0.026	−0.110
F3 Cd	0.014	0.356	**0.894**	−0.106
F3 Cr	0.149	**0.900**	0.249	0.020
F3 Cu	0.178	0.401	0.008	−0.207
F3 Ni	−0.177	**0.934**	0.191	0.066
F3 Pb	0.261	**0.906**	0.107	−0.072
F3 Zn	0.167	**0.890**	0.062	−0.008
F4 As	**0.852**	0.347	0.126	0.042
F4 Cr	**0.909**	0.366	0.074	0.086
F4 Cu	0.450	−0.104	0.000	0.173
F4 Ni	**0.909**	−0.286	0.012	−0.008
F4 Pb	**0.823**	−0.424	−0.187	0.005
F4 Zn	**0.913**	−0.173	−0.245	−0.057
Mortality *H. incongruens*	0.173	−0.512	−0.032	−0.078
Growth inhibition *H. incongruens*	0.530	−0.236	0.105	0.082
Luminescence inhibition *A. fischeri*	−0.090	0.397	0.238	−0.274
Total variance (%)	36.847	20.610	9.054	6.040
Cumulative (%)	36.847	57.457	66.513	72.553

Loadings > 0.6 are shown in bold; *n* = 46

The characteristics of trace elements bound to PC4 indicate their anthropogenic sources (Cd, Ni, Cr) in the Rożnów reservoir bottom sediments. These results are consistent with our previous study, since nickel, chromium and cadmium pose a significant risk in the Rożnów reservoir bottom sediments (Baran et al., 2019b; Szara et al., 2020a). All these elements are deposited in the reservoir mainly with household and industrial wastewater (Vignati et al., 2019). Nickel is widely used in industry, and its high concentrations can be found in sediments and soils in urban areas. Therefore, high nickel content in sediments is a good indicator of recent anthropogenic contamination (de Castro-Catala et al., 2016). As mentioned above, the presence of chromium in the Rożnów reservoir bottom sediments may result from a large number of tanneries operating in the Dunajec river catchment area, having permits to discharge tanning waste water and an unknown number of plants not entered in the register (Vignati et al., 2019). The PCA showed that cadmium behaved differently from other elements determined in the bottom sediments. This relationship was also demonstrated in our previous study on the metal content of core bottom sediment samples from the Rożnów reservoir (Baran et al., 2019b), confirming the anthropogenic sources of the metal. In addition, the significant share of the first three fractions in the Cd bond (exceeding 90% in the present study) and the high bioaccumulation factor (BAFS) value are compatible with the results obtained in various studies on the sediments of many lakes and rivers around the world. They also prove the relatively high mobility of the element in bottom sediments (Njeng et al., 2009; Sundaray et al., 2011; Kulbat & Sokołowska, 2019).

## Conclusions

1. Our study revealed three elements deserving attention in the Rożnów reservoir sediments: cadmium, nickel and chromium. However, cadmium proved to be the most mobile and bioavailable, although the total cadmium content and geochemical indicators did not reveal any risk to organisms. Geochemical indicators showed that the bottom sediments are contaminated with nickel and chromium, but both elements had a low bioaccumulation factor (BASF). Fractional analysis also revealed relatively low mobility of Cr and Ni and a higher potential risk of bioavailability for nickel.
2. Research on the mobility, bioavailability and bioaccumulation of trace elements in bottom sediments is very difficult and requires data on the physicochemical properties of the sediments, as well as the geochemical, ecotoxicological, biological and environmental properties relating to the contamination sources. Only understanding the above factors will provide comprehensive and real, rather than potential, information on the mean effects associated with further activity of trace elements in aquatic systems.
3. Both biotesting and chemical analysis evaluate sediments under standardized conditions which do not necessarily correspond to in situ conditions. Therefore, both can reveal a potential risk to aquatic organisms. However, ecotoxicological tests are more effective in detecting the effects of mixtures of contaminants and substances that are not tested. For a real assessment of the ecological risks associated with trace elements, it is necessary to use bioindicators taken from the environment and exposed to trace elements in situ.

## Declarations

### Human and animals rights

No approval of research ethics committees was required to accomplish the goals of this study.
